# The rate and importance of *Clostridium difficile* in colorectal cancer patients 

**Published:** 2019

**Authors:** Somayeh Jahani-Sherafat, Masoumeh Azimirad, Masoud Alebouyeh, Hadi Ahmadi Amoli, Parnian Hosseini, Hajieh Ghasemian-Safaei, Sharareh Moghim

**Affiliations:** 1 *Department of Microbiology, School of Medicine, Isfahan University of Medical Sciences, Isfahan, Iran*; 2 *Foodborne and Waterborne Diseases Research Center, Research Institute for Gastroenterology and Liver Diseases, Shahid Beheshti University of Medical Sciences, Tehran, Iran*; 3 *Sina Hospital, Tehran Universityof Medical Sciences, Tehran, Iran*; 4 *Department of Pharmacology, University of British Columbia, Vancouver, Canada *

**Keywords:** CRC, Intestinal microbiota, Toxin, *Clostridium difficile*

## Abstract

**Aim:**

The aim of this study was to analyze the *Clostridium difficile* and their toxins in cancerous tissues in comparison to their adjacent healthy tissues in patients with colorectal cancer (CRC) in Iran.

**Background:**

Intestinal infection or colonization by microbial pathogens and their released metabolites may have a role in the exacerbation of CRC.

**Methods:**

A total of 60 biopsy samples from 30 cancerous and 30 adjacent healthy tissues were collected from patients with CRC. Biopsies were homogenized and cultured in cycloserine cefoxitin fructose agar-agar medium to investigate the presence of C. difficile. DNA was extracted, PCR was performed on pure colonies for bacteria detection, and toxin genes were evaluated in each bacterium positive cases. Real-time PCR was performed on extracted DNA for quantitative comparison of *Clostridium difficile* in healthy and tumor tissues in CRC patients.

**Results:**

*Clostridium difficile* was isolated from 18 of the cancerous tissue (60%) and 6 of their healthy adjacent tissue (20%) in the culture medium, but toxin genes were positive just in one sample in both groups. Real-time PCR showed the colonization in all samples.

**Conclusion:**

This study showed a higher prevalence of *Clostridium difficile* in cancerous lesions in comparison to healthy tissues. We suggest that the investigation of the rate of CD of colorectal cancer patients before surgery is critical for patients. Further studies with more samples size to study the importance of this bacterium and its toxins in the investigation of colorectal cancer patients survey is recommended.

## Introduction

*Clostridium difficile*, a gram-positive spore-forming anaerobe, is one of the major concerns in healthcare-associated environments and is the leading cause of antibiotic-associated diarrhea (AAD), colitis, toxic megacolon and pseudomembranous colitis (1, 2). The incidence and severity of Clostridium difficile infection (CDI) has been increased during the past two decades, and about 20-30% of patients with AAD experienced laboratory-confirmed CDI (3).

Colorectal Cancer disease (CRC) is the third-highest cancer morbidity in the world. The main symptoms might include abdominal pain, weight loss, change in bowel habits, bleeding, and anemia. Majority of colorectal cancer cases occur in persons without a family history of colorectal cancer. Although old age is one of the risk factors for colorectal cancer, it seems to be increasing among younger persons (4, 5). Although the pathogenesis of CRC is accurately understood, previous studies confirmed the crucial role of intestinal microbiota on the onset of this disease (6- 8). Antibiotic therapies can alter the typical composition of gut microbiota, which in turn may favor colonization of various pathobionts in the mucosal sites of the intestinal lumen (9).

Several studies have highlighted the individual role of specific bacterial pathogens in exacerbation of CRC (10-12). Some studies have been focused on CDI, shown the association of this infection with excess morbidity and mortality along with with the elevated risk of hospitalization, stop of complementation therapy after surgery and increased systemic costs in CRC patients (13). It was reported that up to 17% of the CRC patients are infected by *C. difficile* (14). Moreover, colonic involvement, chemotherapies, and use of antibiotics reported being as the main risk factors associated with the development of CDI among CRC patients (13).

The majority of commensal microorganisms, collectively known as microbiota that resides in the human body are colonized in niches adjacent to epithelial surfaces of the gastrointestinal tract (15, 16). The diverse and abundant intestinal bacteria play a crucial role in the development and maturation of the immune system early in life, as well as in protection against pathogen colonization (17, 18). However, intestinal infection or colonization by pathogens or a pathobiont, and their released metabolites may alter the composition of the gut microbiota (19, 20). There is limited data regarding the fecal carriage and intestinal colonization of C. difficile among CRC patients. Thus, the main focus of this study was to estimate the prevalence of C. difficile in the gut of Iranian patients with CRC referred to the surgery clinic. 

## Methods

**Patients and sample collection**

Colonic biopsies were collected from 30 patients with CRC, who under surgery for CRC in Bahman Hospital in Tehran from September 2016 to June 2017. All CRC patients had a definite diagnosis based on colonoscopy and pathologic reports. The patients with others organ malignancy or exposed to antibiotic therapy within three months before sample collection, as well as those who had undertaken radiotherapy and chemotherapy before the surgical resection were excluded. 

**Bacterial culture conditions**

The colon biopsies were transported to the laboratory in thioglycolate broth and homogenized with a suitable tissue grinder. Cent microliter of the homogenized biopsy was cultured in the CCFA (under anaerobic conditions at 37^o^C for 48h) for detection of* C. difficile*. The gram-positive isolates with characteristic colony morphology were considered as *C. difficile* isolates and selected for further identification by specific primers (21).

**Total DNA extraction and Polymerase chain reaction (PCR)**

InstaGene matrix extraction kit (Bio-Rad, USA) was used for DNA extraction of *C. difficile* genome (22). Extracted DNA was used as a template for PCR amplification. For molecular identification and confirmation of *C. difficile* isolates, PCR was accomplished by a conserved gene of PaLoc (pathogenicity locus) which is called *cdd3*. For confirming of toxigenic *C. difficile* isolates, PCR was also performed using specific primers for *tcdA*,* tcdB*, *cdtA*, and *cdtB* as described previously (23, 24). The nucleotide sequences of the used primers and PCR process for each PCR assay are shown in table 1.

**Table 1:** Primer sequences and PCR conditions of studied genes

Toxin gene	Primers	Oligonucleotide sequences (5' – 3')	PCR conditions	references	
*cdd3*	Time6 Struppi6	TCCAATATAATAAATTAGCATTCC GGCTATTACACGTAATCCAGATA	94°C 5 min, 40 cycles (94°C 1 min; 53°C 1 min;72°C 45 sec), 72°C 5 min		(23, 24)
*tcdA*	TA1 TA2	ATGATAAGGCAACTTCAGTGG TAAGTTCCTCCTGCTCCATCAA	94°C 5 min, 35 cycles (94°C 1 min; 50°C 1 min;72°C 30 sec), 72°C 5 min		
*tcdB*	TB1 TB2	GACCTGCTTCAATTGGAGAGA GTAACCTACTT CATAACACCAG	94°C 5 min, 35 cycles (94°C 1 min; 50°C 1 min;72°C 30 sec), 72°C 5 min		
16S rRNA gene	27F 1525R	AGAGTTTGATCCTGGCTCAG AAGGAGGTGWTCCARCC	94°C 5 min, 40 cycles (94°C 1 min; 60°C 1 min;72°C 45 sec), 72°C 5 min		(25)
*C.difficile 16srRNA*	C.diff-F C.diff-R	TTGAGCGATTTACTTCGGTAAAGA CCATCCTGTACTGGCTCACCT	94°C 5 min, 40 cycles (94°C 20 sec; 60°C 1 min, 72°C 5 min), 72°C 5 min		(26)

**Figure 1:**
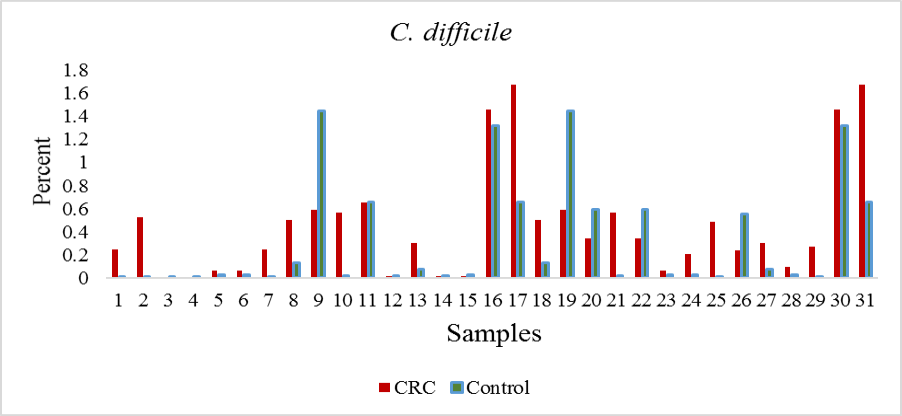
The percentage of *C. difficile* in CRC patients by real time PCR

**Real-Time Quantitative PCR**

To estimate the relative amount of *C. difficile* over the total amount of bacteria, the DNA from each sample was assayed by real-time quantitative PCR (qPCR); the estimation of the total number of 16S rRNA gene copies in all samples was performed with bacterial primers 27F and 1525R targeting the 16S rRNA gene, using a previously reported protocol (25). The value of *C. difficile* was assessed with specific primers which form an amplicon of 151 bp (26), targeting a fragment of the 16S rRNA gene. qPCR was performed in a Rotor-Gene Q apparatus (Applied QIAGEN), amplification program was as follows: 35 cycles of 95 °C for 5 s and 60 °C for 34 s with an initial cycle of 95 °C for 10 min, and a primer pair-specific annealing temperature for 60 s. A melting curve was used to evaluate the presence of primers-dimers. *C. difficile* (ATCC 10898) DNA was used as a standard for qPCR quantification. Reactions were performed in duplicates in 20 μl final volume. PCR results were analyzed by comparing the CT values of the samples, representing the threshold cycles; CT is a relative measure of the concentration of the target gene in the PCR reaction; lower CT values indicate high amounts of targeted nucleic acid, while higher CT values indicate smaller amounts of the target nucleic acid. The presence of *C.difficile* has been calculated as the ratio between the CT value of *C*.*difficile* 16S rRNA gene and the CT value of the total bacterial community 16S rRNA gene amplicons. 

**Statistical analysis**

Data analysis was performed using SPSS software version 21 (SPSS Inc., USA). Statistical differences between the groups were analyzed by T-test, and the results were considered to be significant at a P-value of ≤0.05. The Real-time PCR data were analyzed with one-way analysis of variance (one-way ANOVA) by Prism graft pad soft way. 

## Results

**Patients**

A total of sixty colon biopsies samples from 30 CRC patients were evaluated in this study. Study participants consisted of 14 females (46.6%) and 16 males (53.4%) patients within the age range of 19 to 79 years old and a mean age of 58±12.2 years.

**Bacterial isolates and confirmation with PCR**

**Figure 2:**
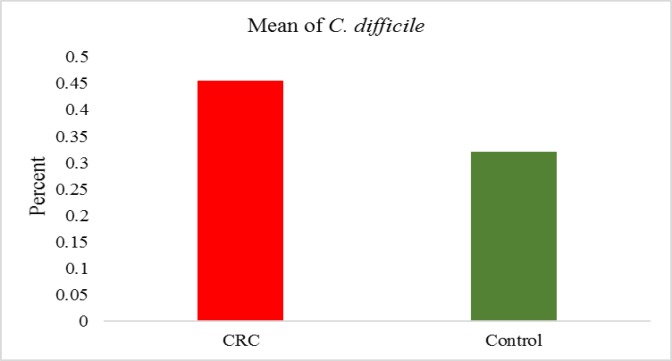
The percentage and average presence of *C. difficile* in cancerous and normal tissues versus total bacteria

*Clostridium* *difficile* was isolated from 24/60 (40%) cases and control samples of 30 patients by culture, 18/30 (60%) positive cultures were belonging to CRC specimens and 6/30(20%) isolates from healthy tissues. This difference was statistically significant (P=0.044). All isolates were confirmed by PCR and were positive for *cdd3 *genes. Just two of the isolates were positive for toxins and were encoded by *tcdA*, *tcdB,* and both positive toxins belong to one patient in its healthy and tumor tissues. But no one was positive for binary toxin genes.

**Real-time PCRs**

The quantification of *C. difficile *expressed as the ratio between the CT value of *C. difficile *16S rRNA gene, and the CT value of the total bacterial community 16S rRNA gene amplicons is reported. The Real-time PCR showed all of the samples were positive for *C. difficile*. Consequently, the ratio values indicate higher *C. difficile *abundance in the tumor tissue samples (figure 1 and 2). But no significant differences between tumor samples and healthy tissue were observed CRC patients (p<0.076). 

## Discussion

CRC is becoming an emergent disease in the developed and recently developing countries, in a relatively short period. CRC is disabling for young patients, generating a substantial burden on health-care systems in the world (12). Lifestyle is an important issue in impairing of the microbiota of the human gastrointestinal (27). The interplay of microbiota with immune systems has a significant effect on instruction and regulation of the mucosal immunity. Excessive and dysregulation of the mucosal immune response in CRC patients can be linked to abnormal and abrogated microbial communities (Dysbiosis) in the gut of CRC patients (28). A lack of diversity of the gut microbiome and colonization of pathogenic bacteria can be reasons of dysbiosis (29). 

In recent studies, showed patients, who cured with broad-spectrum antibiotics, hospitalized and immunocompromised, are at increased risk for the CDI. Because of the presence of the same these risk factors in CRC Patients, CDI can quickly be developed in CRC patients (30). The accurate colonization role of *C. difficile* in CRC patients has not been determined until now. *C *difficile can produce some toxins (enterotoxin A, cytotoxin B, and binary toxin), which can initiate an inflammatory response in the colon (31). Chronic inflammatory could be one of the initiation pathways toward the CRC by DNA damage (32). 

In this study, 60 CRC patients were introduced for assessment of *C. difficile* colonization. Forty percent of samples in this study were positive for *C. difficile* with culture methods, whereas 100% of samples were positive with real-time PCR but with different percentage. *C. difficile* is identified as the most common bacteria in the colon. Pathogenesis of *C. difficile* is dependent on toxin production, and the toxins are a crucial role in pathogenicity. In our study, Rate of toxins positive *C. difficile* isolates was low (3.3%) but, other studies have been stated higher percent of toxigenic *C. difficile* in CRC patients (14). One reason for this observation may be related to our method, because isolates from culture were an examination for toxin, while *C. difficile* were positive in all the samples by Real-time PCR. Zheng et al. showed 16.1 percentage of preoperative CRC patients were *C. difficile* positive with 19% toxigenic* C. difficile* (14). However, in contrast to our results, a study reported rate of C. difficile colonization in admitted children in the hematologic ward was reported to be 25.6%, with a 92.6% of toxigenic strains (33). The previous study revealed 20.5% of toxigenic *C. difficile* colonization in cancer patients, and they concluded that CDI risk could increase 4.8-fold in cancer patients (34). Several studies suggested that more generally colon involvement, are risk factors for CDI and the risk of developing CDI is more in post-surgery cancer patients (14, 33) So, screening of *C. difficile* for every patient with colon complication and risk factors was recommended. This fact shows a requirement of a rapid test for CDI detection and starts an appropriate treatment promptly.

The impact of *C. difficile* on CRC is not well clear, but Patients admitted to hospital with CRC have many of these risk factors and may be predisposed to *C. difficile*. Our results elucidate that 100% of CRC patients were *C. difficile* positive. In CRC patients, it has been described that CDI can increase morbidity and mortality rate in post-surgery infected. So, early detection and treatment CDI is important and problematics issue in CRC patients, but more studies are needed to determine the risk factors causing the transformation from *C*. *difficile* colonization to CDI in CRC patients.

*C. difficile* is a common bacterium in the Colon of CRC patients but, after the hospitalized and the treatments which induce immunodeficiency the occurrence of CDI in CRC patients have been scarcely explored. Also, antibiotic resistance can challenge the treatment of CDI in CRC patients in the future. So, *c. difficile* monitoring is a crucial issue before starting chemotherapy and radiography in CRC patients.

## Conflict of interests

The authors declare that they have no conflict of interest.
